# Alport syndrome misdiagnosed with IgA nephropathy from familial history: a case report and brief review

**DOI:** 10.1186/s12882-023-03165-7

**Published:** 2023-04-15

**Authors:** Hormat Rahimzadeh, Sanaz Ajlou, Fatemeh Nili, Effat Razeghi

**Affiliations:** 1 Department of Nephrology Diseases, Sina Hospital, Tehran University of Medical Sciences, Hasan Abad Sq, Tehran, 11367-46911 Iran; 2 Department of Internal Medicine, Sina Hospital, Tehran University of Medical Sciences, Tehran, Iran; 3 Department of Pathology, Imam Khomeini Hospital Complex, Tehran University of Medical Sciences, Tehran, Iran; 4 Nephrology Research Center, Center of Excellence in Nephrology, Tehran University of Medical Sciences, Tehran, Iran

**Keywords:** Alport Syndrome, IgA Nephropathy, Glomerular Disease, Hematuria, Proteinuria, Renal Biopsy, Case report

## Abstract

**Background:**

Alport syndrome is a rare inherited disease resulting from a primary disorder of the glomerular basement membrane. This disease results from mutations in genes encoding alpha chains of type IV collagen. In the differential diagnosis of this disease, IgA nephropathy is the most common primary glomerular disease with gross or microscopic hematuria.

**Case presentation:**

A 50-year-old woman was presented with microscopic hematuria and proteinuria of under one gram. Due to the diagnosis of IgA nephropathy in family members, she was treated and followed up for 4 years as a possible case of IgA nephropathy. Eye examination and audiometry were normal. She underwent renal biopsy with an exacerbation of proteinuria. There was no finding in favor of IgA nephropathy in the histological examination, but the findings of electron microscopy and family history favored Alport syndrome.

**Conclusions:**

This case demonstrates the importance of accurate history and electron microscopy in the complete histological evaluation and diagnosis of glomerular disease. Although in most cases the two can be differentiated based on clinical manifestations, laboratory findings, and histopathological examination, sometimes the association of these two diseases in the families involved or the lack of accurate history and complete histological examinations can complicate the diagnosis.

## Background

Alport syndrome, also known as hereditary nephritis, is a progressive inherited glomerular disease caused by a disorder of α3, α4, and α5 chains in type IV collagen [1]. This rare glomerulopathy is the cause of 3% of CKD in children and 0.2% of all ESRD in adults and is often associated with hearing loss and visual impairment. Pathogenic mutations in COL4A5 and mutations of COL4A3 and COL4A4 are transmitted through X-linked and autosomal patterns, respectively [2]. Coexistence of Alport syndrome with IgA nephropathy and even ANCA vasculitis has been reported as some case reports [3, 4]. Due to the rarity of this disease, a high rate of misdiagnoses have been seen, especially in cases where there are no extrarenal manifestations. We report a 50-year-old female with Alport syndrome who was misdiagnosed with hematuria and proteinuria and followed up with a possible diagnosis of IgA nephropathy due to reports of IgA nephropathy in family members.

## Case presentation

The patient was a 50-year-old female hospitalized in our center due to edema, microscopic hematuria, and increased proteinuria. She had no problems until she was diagnosed with hematuria and proteinuria of less than one gram per day in a routine test 4 years ago and was treated with Losartan and Atorvastatin with a possible diagnosis of IgA nephropathy. Although she had no regular follow-up during this time, she did not report any particular complaints. Only a few months ago, she developed lower extremities edema, and tests showed that proteinuria had increased to more than one gram, so she was referred to our center for a biopsy and further examination. The patient did not report any particular complaints at the clinic, except for the swelling of both lower limbs below the knee, which had developed and gradually increased in recent months.

Medications used included Atorvastatin, Losartan, and Pentoxifylline. The patient’s father had no problems in the family history, and her mother was deceased, but there was no other information about her mother’s medical records except the history of high blood pressure. This patient had four sisters, and five brothers, 3 of whom had kidney failure, and 2 of them underwent kidney transplantation under the age of 30. The two had undergone kidney biopsy, but the findings of one case were not conclusive, and for the second case, IgA nephropathy was reported based on histopathological findings and diagnostic IF. She (our patient) also had five children, and all were boys; two of whom underwent kidney transplants under the age of 30. One of the two children underwent a pre-transplant kidney biopsy, which reported IgA nephropathy. However, the latter was not a candidate for biopsy due to the small size of his kidneys at the time of diagnosis of renal failure. At the time of hospitalization, the patient had BP = 120/80 mmHg, and a temperature of 37° C. Physical examinations were negative in other respects except for edema, which was 2 + in the lower extremities. Laboratory tests included CBC, platelets, BUN, creatinine, electrolytes, liver tests, thyroid hormones, serum lipids, complement, ANA, ANCA, and viral markers were normal. Urine analysis showed blood 4 + , with many RBCs, and protein of 3 + . The 24-h urine collection contained 1,542 mg of protein. On sonography, it was reported that the size of the kidneys was normal, with a slight increase in the echoes of both kidneys; in addition, a 11 mm cortical cyst was located in the left kidney. Eye examination and audiometry were unremarkable. A kidney biopsy was performed to assess renal involvement severity and rule out other possible diagnoses. In addition to previous medications with a possible diagnosis of IgA nephropathy and due to family history, Prednisolone was started at a dose of 1 mg/kg body weight, and it was recommended to return with histopathological results.

**Fig. 1:**
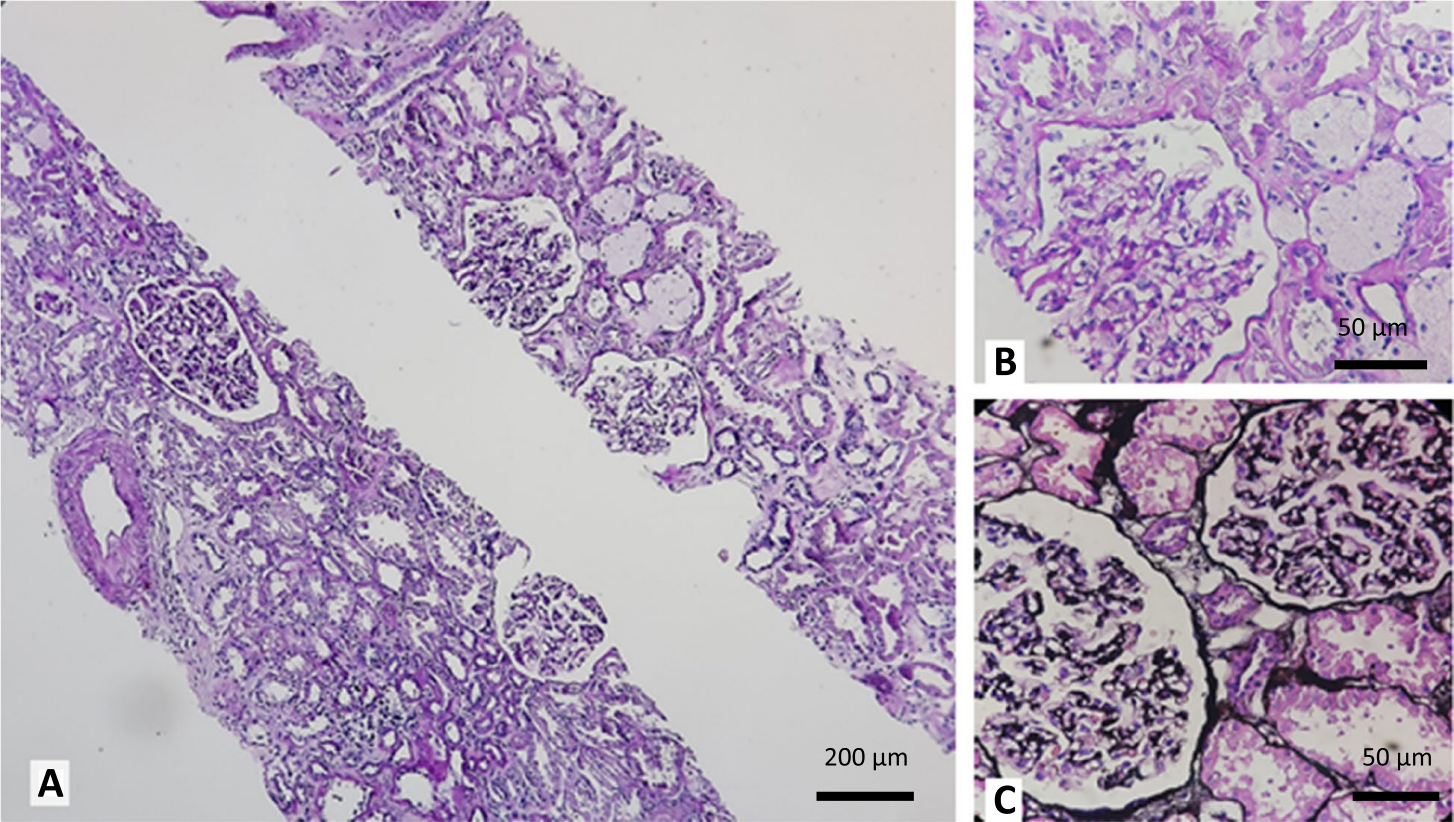
Microscopic examination of Hematoxilin and Eosin, PAS and Jone’s stains shows normal appearing glomeruli, aggregates of foam cells in the interstitium, mild interstitial fibrosis and tubular atrophy as well as mild atherosclerosis (**A**: 100X, **B**, **C**: 400X)

**Fig. 2:**
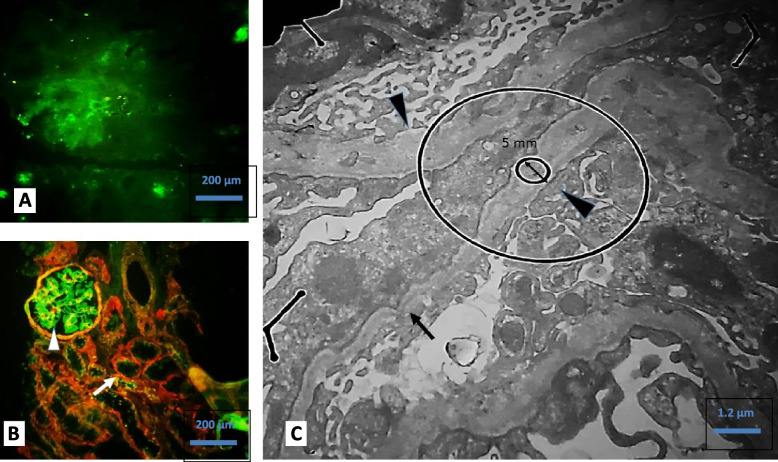
**A** Immunofluorescence study for IgG, IgA, IgM, C3c, C4c, C1q, Kappa and Lambda shows negative reaction in glomeruli and tubulointerstitium (100X). **B** Immunofluorescence study for α2 chain of collagen type IV (red fluorochrome) shows normal staining pattern (arrow). α5 chain of collagen type IV (green fluorochrome) shows weak staining in GBM and distal tubule basement membrane (arrow head) (100X). **C** Ultrastructural evaluation by TEM study shows irregular GBM with alternating foci of thinning (arrow) and thickening (arrow head) (8000X)

**Fig. 3:**
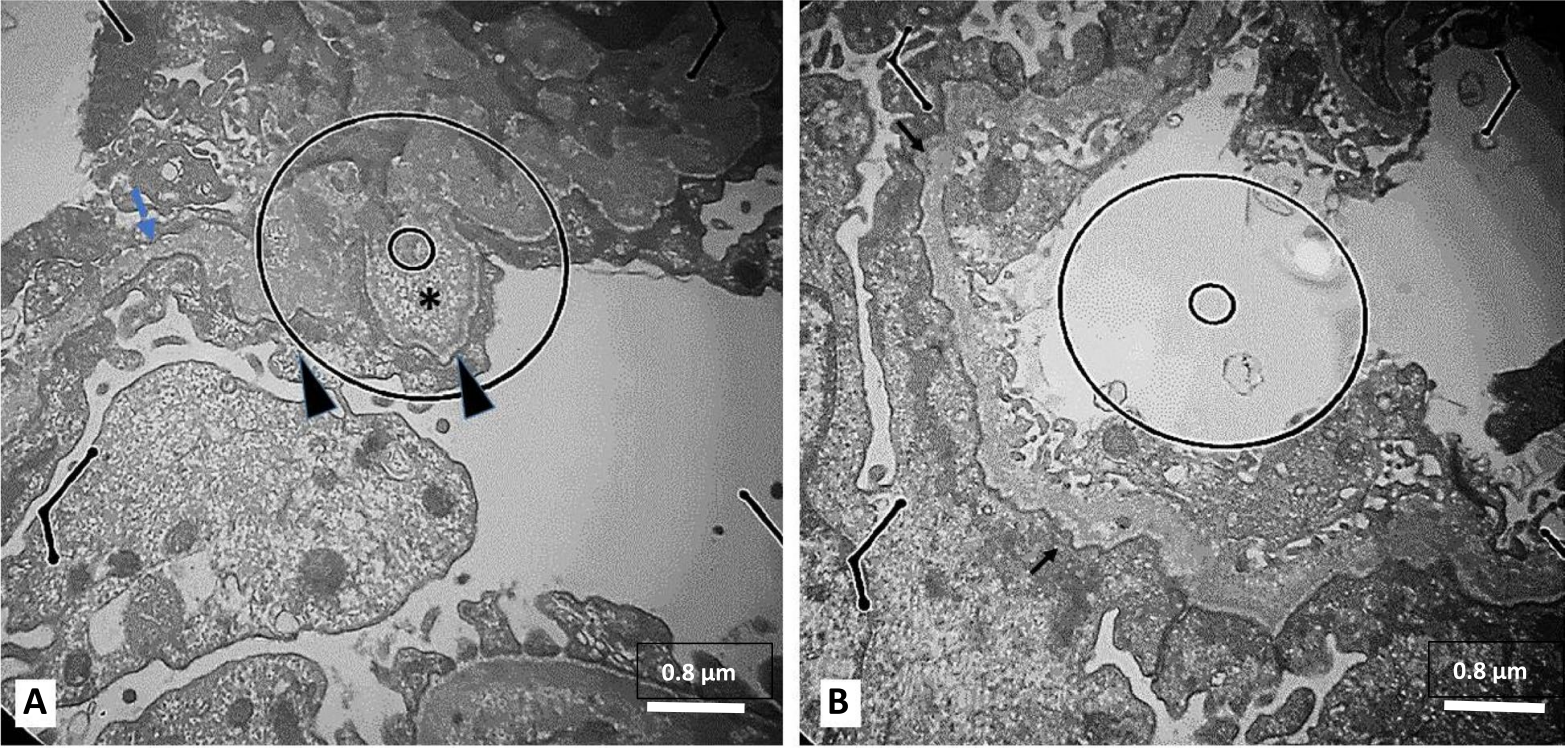
**A**, **B** Higher magnification of the GBM shows alternating thinning (blue arrow) and thickening (arrow heads) of GBM with multilayering of lamina densa making basket weave appearance (star) and scalloping at epithelial side (black arrows) (11000X)

The patient’s tissue biopsy results were reported as follows: on LM and IF studies, no significant pathologic finding identified. However on electron microscopy, GBM changes including multilayering of lamina densa and irregular thickness were suggestive of hereditary nephritis (Alport syndrome). Neither IgA deposit on IF study nor electron dense deposits on EM were seen (Figs. 1, 2 and 3).

Due to the high cost and lack of access in our center, genetic testing was not possible. Also, in our center, the only examination that was possible was evaluation of the five chains of collagen IV in the kidney biopsy specimen, which was performed for the patient, and no abnormality was reported. According to the pathology report, the medical history of the family members were re-examined, and it was found that one of her children and her brother had severe hearing loss in the course of the disease. Furthermore, the daughter of one of her brothers (He had been diagnosed with IgA nephropathy), underwent a kidney biopsy at the age of four due to hematuria and proteinuria, and histology and electron microscopy findings favored Alport syndrome.

## Discussion

Alport syndrome is a rare inherited disease that should be distinguished from IgA nephropathy, the most common primary glomerular disease affecting 2.5 per 100,000 people worldwide [5, 6], especially in older patients with unexplained hematuria and proteinuria in the absence of extrarenal symptoms. Having a family history of kidney disease with a diagnosis of IgA nephropathy, the patient was treated with the possibility of familial IgA nephropathy. However, the family was not thoroughly evaluated for Alport syndrome, while IgA nephropathy with Alport syndrome has been reported [3]. Alport Syndrome is a genetically heterogeneous disease caused by genes encoding alpha 3, 4, and 5 chains of IV collagen [1]. These IV collagen alpha chains are naturally located in the basement membrane of the kidneys, cochlea, and eyes. The disruption in these chains leads to the defect of the basement membrane in these areas and ultimately to the clinical symptoms of the disease. The disease is genetically transmitted in three types of autosomal dominant, autosomal recessive and X-linked forms, with the most common form of transmission being X-linked (85%), caused by a mutation in the COL4A5 gene on the X chromosome. The recessive form is the reason of 15% of cases and is caused by homozygous or compound heterozygous mutation, COL4A3 or COL4A4, and the predominant form is seen in less than 5% of cases and is caused by heterozygous mutation COL 4 A3 or COL 4A4. However, digenic inheritance is seen due to mutation transmission in two of the three genes in some families.

Furthermore, next-generation sequencing reports in Alport families suggested that the autosomal dominant form may be more common (20–30%) than expected [7, 8]. The X-linked and autosomal recessive forms have the same clinical and pathological features, and both cause renal dysfunction in early childhood. Patients with the autosomal dominant disease have a slower course and reach the ESRD later [9, 10]. Hearing and visual impairment is also common in autosomal recessive and X-linked forms, while these disorders are uncommon in the autosomal dominant condition. A family history of ESRD with hearing and visual impairment helps diagnose Alport syndrome, but the diagnosis is made by molecular genetic analysis or kidney and skin biopsy. Of course, the preferred and noninvasive method for diagnosis is molecular genetics, provided that the cost of the agent and unavailability are not limiting. Genetic testing can confirm early diagnosis of the disease and has high sensitivity and specificity[11], but we could not perform this study due to high cost and lack of access in our center.

Histological lesions include GBM thinning and varying GBM segmental thickening and splitting caused by alpha chains of type IV collagen mutations. In many cases, a basket weave is seen with electron microscope instead of the typical ribbon-like morphology. These structural disorders of GBM cause its dysfunction and pathophysiological signs, including chronic inflammation and eventually fibrosis [12]. A renal presentation of Alport syndrome is initially asymptomatic microscopic hematuria, although sometimes gross hematuria following an upper respiratory infection can be an early finding. Blood pressure and kidney function are normal at first, but hypertension, exacerbation of proteinuria, and progressive renal failure occur over time, and patients progress to ESRD between the ages of 16 and 30, with the course of the disease being slower in women. Alport syndrome is typically differentiated from other significant causes of persistent glomerular hematuria, with a family history of hematuria, along with renal failure and deafness. Glomerular diseases that occur with microscopic hematuria, including IgA nephropathy and thin basement membrane nephropathy, are included in the differential diagnosis of Alport.

In IgA nephropathy, although a family history of bilateral hearing loss has been reported, it is not commonly seen [13]. A family history of hematuria can be positive in thin basement membrane disease, but kidney failure and deafness are not commonly seen or occur relatively late. For this reason, in recent years, this disease has been considered a variant of Alport. The patient was diagnosed with hematuria and proteinuria but had no ocular or auditory problems, although the absence of sensorineural hearing loss should not reduce the likelihood of Alport syndrome in the differential diagnosis. Most women with CKD due to X-linked Alport syndrome have no hearing loss and fewer of them compared with men progress to ESRD [14]. This patient had a family history of kidney disease with IgA nephropathy in family members, which led to a possible diagnosis of familial IgA nephropathy. Although family members were not thoroughly evaluated, such as electron-microscopic tissue examination and immunostaining for alpha-chain disorder and genetic studies, IgA nephropathy with Alport syndrome has been reported [3]. Another factor that led to the diagnosis of IgA nephropathy was the presence of familial IgA nephropathy with bilateral hearing impairment [13], although this is not common. However, the clinical course of kidney involvement in family members, hearing impairment, and severity of involvement in boys were more consistent with Alport syndrome, which unfortunately was not given enough attention. On the patient’s renal biopsy, mild histopathological changes were reported with an adverse immunofluorescent reaction that ruled out IgA nephropathy. Electron microscopy findings included GBM segmental thickening and thinning and multilayering, longitudinal splitting of lamina densa into capillary walls favoring hereditary nephritis (Alport syndrome). Only immunostaining was performed for the alpha five chain, which had no abnormality given the available facilities. In some patients with Alport syndrome, whose exact mechanism is unknown, the collagen IV alpha chain may not be abnormal. Some mutations may have a more negligible effect on outcome and performance [3].

According to the findings, Alport's inheritance in this family is probably X-lined, although studies are not complete and definitive. Treatment of IgA nephropathy can vary depending on the degree of proteinuria and the severity of the kidney involvement, while there is no specific treatment for Alport syndrome. Renin-angiotensin inhibitors are started to control urinary protein in protein-creatinine ratio > 0.2, and also maintenance measures are taken to prevent and treat the complications of chronic renal failure and eye and ear involvement. However, in uncontrolled studies in humans, different results have been reported with cyclosporine in Alport [15]. In the early years, the patient was treated with Losartan, Atorvastatin, and Pentoxifylline, which appears helpful in most glomerulopathies. Nevertheless, after performing a kidney biopsy and before receiving tissue results, she was placed on 1 mg/kg Prednisolone for one month. Although proteinuria fell below one gram, the patient did not continue treatment due to weight gain and muscle weakness. Therefore, the definitive effect of prednisolone cannot be commented on in this patient. Also, our center did not have patient follow-ups due to the Corona pandemic and distance problems. In the ESRD patients, transplantation is a good choice with excellent results, and recurrence of the disease after transplantation does not occur due to the donor usually having normal GBM, but the anti-GBM disease can ensue and has been reported [16]. A single Korean center also reported a higher incidence of BK virus nephropathy after transplantation in children with Alport [17]. Therefore, it is essential to correctly diagnose Alport syndrome and differentiate it from other glomerulopathies in the proper care of patients before reaching the stage of advanced renal failure and pay attention to the problems related to this disease after kidney transplantation. Genetic disease monitoring in pregnancy is also useful in helping families with appropriate measures. However, we had limitations in diagnosing the genetic disorder due to the inability of genetic testing and proper care for her and her family members owing to distance problems and the Corona pandemic.

## Conclusion

The diagnosis of Alport disease and its differentiation from common IgA nephropathy require careful attention and sometimes a frequent review of family history as well as a thorough histological examination, especially electron microscopy, in suspected cases. In addition, a prolonged follow-up seems to be necessary to detect the delayed onset of symptoms. Although there is currently no specific treatment other than supportive measures for this syndrome, the administration of prednisolone reduced proteinuria in this patient. Therefore, the benefits of treatment need further study.

## Data Availability

The patient data during the current case report are available from the corresponding author on reasonable request.

## Abbreviations

IgA: Immunoglobulin A
CKD: Chronic kidney disease
ESRD: End stage renal disease
COL4: Collagen type 4
CBC: Complete blood cell
BUN: Blood urea nitrogen
RBC: Red blood cell
ANA: Antinuclear antibody
ANCA: Antineutrophil cytoplasmic antibody
LM: Light microscope
IF: Immunofluorescence
EM: Electron microscope
GBM: Glomerular basement membrane
